# Examination of the association between temperature variability and cardiovascular and respiratory mortality in South Africa, 2006–2016

**DOI:** 10.3389/fepid.2025.1553553

**Published:** 2025-06-03

**Authors:** Malebo Sephule Makunyane, Hannes Rautenbach, Janine Wichmann

**Affiliations:** School of Health Systems and Public Health, Faculty of Health Sciences, University of Pretoria, Pretoria, South Africa

**Keywords:** temperature variability, cardiovascular disease, respiratory disease, mortality, time-series, DLNM, South Africa

## Abstract

**Background:**

Evidence is limited on the impact of temperature variability (TV) on health in low-and-middle-income countries (LMICs), such as South Africa. This study examined the association between TV and cardiovascular disease (CVD) and respiratory disease (RD) mortality in five South African cities.

**Methods:**

Daily mortality and meteorological data in five South African cities (Bloemfontein, Cape Town, Durban, Johannesburg, and Gqeberha) were collected from Statistics South Africa and the South African Weather Service for the period 2006–2016. TV was calculated as the standard deviation of the daily minimum and maximum temperatures over the exposure period. City-specific risks were estimated using quasi-Poisson regression models combined with distributed lag nonlinear models, adjusting for potential confounders. A meta-analysis was then conducted to pool the overall estimates across cities. Additionally, stratified analyses by age group and sex were performed to assess effect modification.

**Results:**

A total of 213,875 cardiovascular and 114,887 respiratory deaths were recorded in the five cities during the study period. The risks with increasing TV were higher for RD mortality as compared to CVD mortality. The pooled estimates showed the highest and significant increase in RD mortality of 1.21(95% CI: 1.04;1.38) per an increase in TV at 0–2 days from the 25th to the 50th percentile for all ages combined. The elderly appeared more vulnerable to RD mortality than <65 years age group, with significant mortality risks per increase in TV at 0–2 days (RR = 1.18, 95% CI: 1.04; 1.32),0–3 days (RR = 1.16, 95% CI: 1.04; 1.28) and at 0–7 days (RR = 1.12, 95% CI: 1.02; 1.22) from the 50th to the 75th percentile. A stratified analysis showed the elderly and women as more vulnerable. The pooled results across the five cities suggested no statistically significant TV effect on CVD mortality.

**Conclusion:**

This study found a short-term association between temperature variability and respiratory mortality, especially among elderly individuals and women, in five South African cities. No significant effect was observed for cardiovascular mortality. The findings support targeted public health strategies that account for temperature-related risks in vulnerable populations.

## 1 Introduction

Non-communicable diseases (NCDs) are globally regarded as a cause of premature death (1, 2). Cardiovascular and chronic respiratory diseases are among the top five NCDs that were prioritized to reduce the global burden of mortality and morbidity at the third United Nations High-Level Meeting on NCDs (3). A total of 71% of deaths that occur globally are attributable to NCDs, with 85% of these deaths occurring in low- and middle-income countries (LMICs) due to poor infrastructure and inadequate adaptation and mitigation strategies (4–7). In 2017, 57.8% of all deaths in South Africa were attributed to non-communicable diseases (NCDs). Among these, diseases of the circulatory system [classified under codes I00–I99 of the International Classification of Diseases, 10th Revision (ICD-10)] accounted for 18.4% of all deaths, while diseases of the respiratory system (ICD-10 codes J00–J99) accounted for 9.5% (8).

Most global climate-related studies have focused on the health impacts of increases in mean temperatures, paying relatively little attention to the health effects of temperature variability (TV), which refers to short-term fluctuations in ambient temperature (5, 9, 10). Climate change not only causes an increase in average temperatures but also leads to greater fluctuations in temperature with its associated extremes (10–12). Climate variability refers to the natural variations in climate patterns that occur over time, both on a regional and global scale. It includes variations in meteorological variables such as temperature, rainfall, and wind patterns (13). Natural factors, such as changes in solar radiation or volcanic activity, or anthropogenic factors, such as land use or greenhouse gas emissions, are both considered as drivers of climate variability, and eventually, climate change (13, 14).

To date, the impact of TV indicators on health outcomes in South Africa had been investigated in only two time-series epidemiological studies (15, 16). In the first study, the effects of various meteorological variables, including diurnal temperature range (DTR), on hospital admissions due to pneumonia in two government public hospitals in the Limpopo Province were investigated. The results indicated that an increase in pneumonia hospitalizations is significantly associated with an increase in DTR (an indicator of TV) (16). In the second study, the health effects of the composite index of inter- and intraday TV on CVD and RD hospital admissions in private hospitals in Cape Town were investigated. The researchers observed significant associations between an increase in TV and an increase in hospital admissions, even after controlling for several confounders (15). The limitations of these studies include their region-specific focus, relatively small sample sizes, and shorter study periods, which limit the generalizability of the findings to other parts of South Africa. Furthermore, one study used DTR as an exposure variable, which is another limitation as a multi-country study ascertained that the effects of TV last for several days before exposure which is not captured by DTR (10). Therefore, using an inter- or intraday TV index (such as DTR) might underestimate the risks of TV on health outcomes. While DTR captures within-day temperature variation, it does not reflect between-day fluctuations or the cumulative impact of temperature variability over several days. These warrant more epidemiological studies assessing health risks associated with climate change and variability in South Africa.

Consequently, the present study aims to extend the current knowledge on the health effects of climate variability and change in South Africa by examining the association between TV and CVD and RD mortality in five South African cities (namely Bloemfontein, Cape Town, Durban, Johannesburg, and Gqeberha). Secondly, stratification by age and sex was performed to identify potential effect modifiers of the association between TV and mortality.

## 2 Methods

### 2.1 Data collection

**Figure 1:**
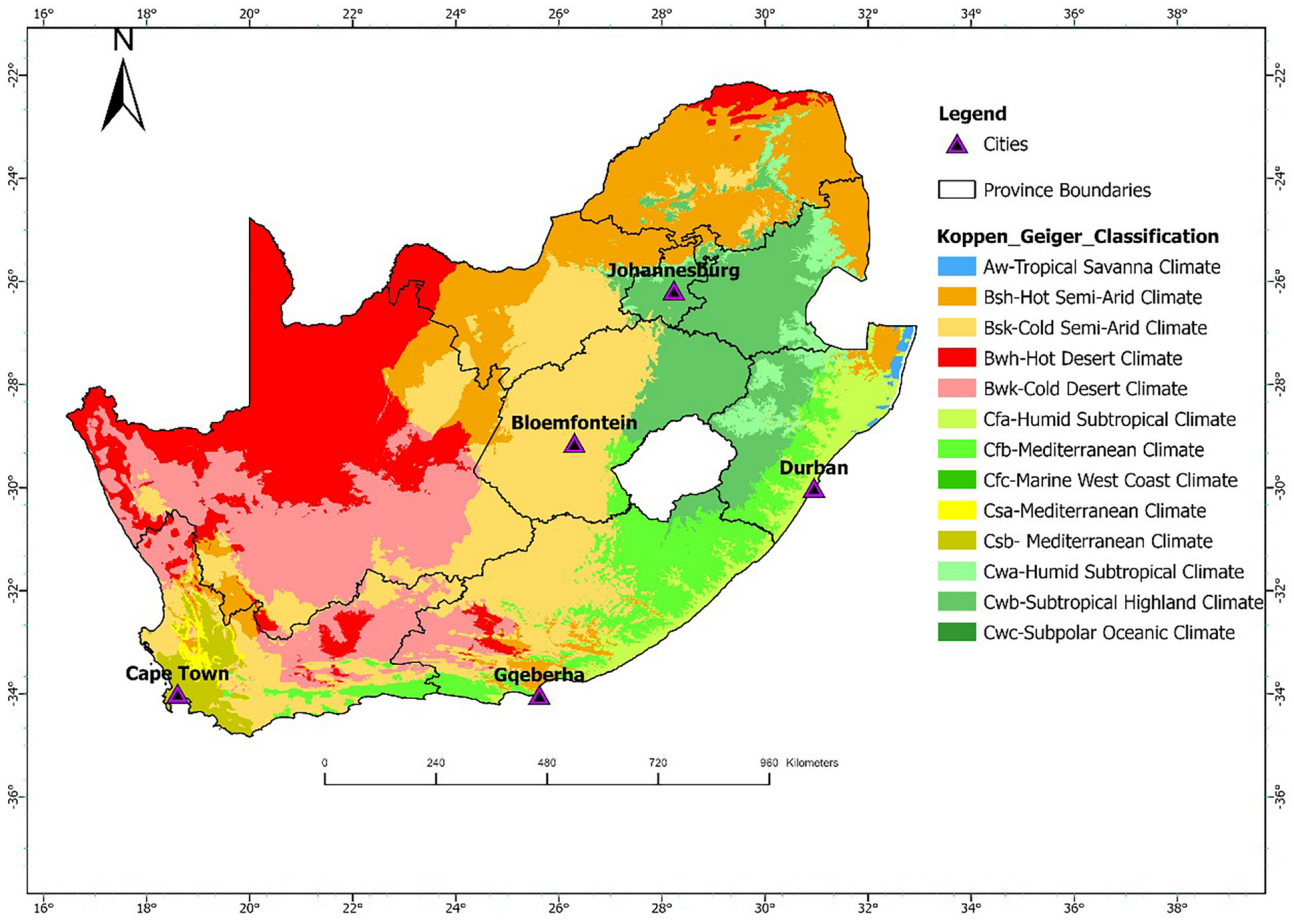
Geographic locations of Cape Town, Bloemfontein, Durban, Johannesburg, and Gqeberha in South Africa. The link for the Koppen Geiger Classifications shapefile were downloaded from http://stepsatest.csir.co.za/climate_koppen_geiger.html and the boundary shape file was acquired from the Municipal demarcation Board of South Africa (https://dataportal-mdb-sa.opendata.arcgis.com/).

Daily count data of CVD and RD deaths for the five cities were obtained from Statistics South Africa (Stats SA) for the 11-year study period from 1 January 2006 to 31 December 2016. The mortality data were classified using the International Classification of Diseases, 10th Revision (ICD-10), based on the primary cause of death: RD (ICD-10: J00–J99) and CVD (ICD-10: I00–I99). All deaths, whether occurring in hospitals or in the community are legally required to be registered. Cause-of-death information is typically certified by a medical practitioner, or in the case of non-hospital deaths, by forensic or traditional practitioners. Stats SA codes and manages death records through its national civil registration and vital statistics system. While national coverage of death registration is relatively high (>90%), some variation in the completeness and accuracy of cause-of-death reporting may exist across regions (8). Meteorological data for the five cities were obtained from the South African Weather Service (SAWS) and included hourly temperature (°C) and relative humidity (%), recorded at ground-based weather stations. These stations were primarily located at major airports near the urban centers and were selected based on their proximity to the city (within 20 km) and the completeness of their data records. The locations of these stations are displayed in Figure 1.

### 2.2 Study design and location

For this study, a time-series epidemiological approach was followed. As a study area, five South African cities located in different Köppen-Geiger climatic zones were included: Bloemfontein, Cape Town, Durban, Johannesburg, and Gqeberha. These cities represent a range of climatic conditions across the country. Bloemfontein falls within the BSh zone (hot semi-arid), characterized by low annual rainfall and hot summers. Cape Town is classified as Csb (warm-summer Mediterranean climate), with dry, warm summers and cool, wet winters. Durban lies in the Cfa zone (humid subtropical climate), marked by hot, humid summers and mild, moist winters. Johannesburg is situated in the Cwb zone (subtropical highland climate with dry winters), experiencing warm summers and cool, dry winters. Gqeberha (formerly Port Elizabeth) lies in a transitional zone, primarily Cfb (temperate oceanic climate), with relatively uniform rainfall throughout the year and mild temperatures. The geographic location of these cities is shown in Figure 1. These cities were also selected since they are the most populated cities in the country. They are therefore representative of the different climatic conditions experienced in South Africa with adequate data to justify the research.

### 2.3 Exposure definition

Temperature variability (TV) refers to short-term fluctuations in ambient temperature that may exert additional stress on human health, beyond the effects of mean or extreme temperatures alone. Multiple definitions of TV exist in literature, depending on data availability, temporal resolution, and the epidemiological context (10, 17, 18). Consistent with Guo et al. (10) we defined TV as the standard deviation (SD) of the daily minimum and maximum temperatures over a given exposure window to capture both intra- and inter-day variation. For instance, TV at 0–1 day was calculated as the SD of the daily minimum and maximum temperatures on the day of the health outcome and the preceding day. Similarly, TV at 0–2 days included data from the day of the event and the two preceding days, and so on, up to a 7-day exposure window (TV 0–7).

### 2.4 Statistical analysis

Previous studies have often assumed a linear relationship between temperature variability (TV) and health outcomes (10, 18, 19). In contrast, our model comparison using Akaike's Information Criterion (AIC) supported a non-linear distributed lag structure, indicating that the association between TV and mortality was non-linear and exhibited delayed effects in all five cities. To estimate these associations, we employed a two-stage analytical approach, combining a distributed lag non-linear model (DLNM) with a quasi-Poisson generalized linear model to account for overdispersion in mortality counts. This modeling strategy has been widely applied in multi-city and multi-country time-series investigating temperature-related health effects, such as hospital admissions or mortality (10, 20, 21).

In the first stage, city-specific estimates of CVD and RD mortality risks were obtained using the following model:

$$E(\log\;(\mathrm{Mor}t_{i})=\alpha +cb(TV_{0-i})+cb(\mathrm{Tmea}n_{t,i})+ns(\mathrm{tim}e_{i},11\times df)+s(\mathrm{RH},3)+\lambda \mathrm{do}w_{i}\times \mathrm{sint}\lambda +\mathrm{do}w_{i}\times \mathrm{cost}+\delta \mathrm{pu}b_{i}$$

Mort indicates the daily count of CVD or RD mortality in a city, while $cb(TV_{0-i})$ denotes the cross-basis matrix of TV and its lag structure in the DLNM, which differed from city to city and health outcome (CVD or RD). Refer to the appendix (Supplementary Tables A1 and A2) for details of the specifications of the cross-basis function for each city. Three internal knots were placed at equally spaced TV percentiles (25th, 50th, and 75th) and for the lag structure, two internal knots at log values plus an intercept. The median (50th percentile) was selected as the reference value of the TV exposure variable to calculate the relative risks. The TV mortality association was reported at the 25th percentile, 75th percentile and 99th percentile relative to the reference value. $ns(\mathrm{tim}e_{i},11\times df)$ denotes a natural cubic spline for calendar time to control for long-term and seasonal trends with *df* degrees of freedom for each year in the study period. The degrees of freedom ranged from four to eight for CVD models, and from five to nine for RD models. s(RH,3) denotes the natural cubic spline function applied to relative humidity (RH) with 3 degrees of freedom, allowing for a flexible, non-linear relationship between RH and the health outcome $cb(\mathrm{Tmea}n_{t,i})$ denotes a cross-basis function to account for the delayed and nonlinear effects of daily mean temperature. Similar to previous studies, a natural cubic spline with four degrees of freedom was used for the daily mean temperature and to capture the lags over time up to 21 days (10, 18, 21). The models included an indicator variable for the day of the week $\left(\mathrm{do}w_{i}\right)$ and public holidays (pub) which considered public holidays as well as other important events (e.g., election days).

Additionally, to account for potential variations in the day-of-the-week (dow) variables due to seasonal fluctuations, the model included cost and sint variables with yearly cycles. These variables were supplemented with interaction terms involving the day-of-the-week variables [Equations (1) and (2)] (22).

(1) $$\mathrm{cost}=\cos\;\left(\frac{\mathrm{time}\times 2\times \pi }{365.25}\right)$$

(2) $$\mathrm{sint}=\sin\;\left(\frac{\mathrm{time}\times 2\times \pi }{365.25}\right)$$

To obtain pooled effect estimates for the association between temperature variability (TV) and cause-specific mortality, we conducted a two-stage meta-analysis. In the first stage, city-specific relative risks (RRs) and 95% confidence intervals (CIs) were estimated for each TV exposure window (0–1 to 0–7 days) and TV percentile level (25th, 75th, and 99th), using the 50th percentile as the reference. A fixed-effect model was used when between-city heterogeneity was statistically insignificant (Cochran's *Q* test *p* ≥ 0.1), while a random-effects model was applied when heterogeneity was present (*p* < 0.1), thereby accounting for differences in local contexts across the five cities. Additionally, to explore potential effect modification, stratified meta-analyses were conducted by age group (<65 years and ≥65 years) and sex (females and males).

Sensitivity analyses were conducted to evaluate the robustness of the city-specific model estimates. First, we extended the lag structure of the temperature cross-basis function from 21 to 28 days to assess whether the selected lag period sufficiently captured delayed temperature effects. Second, we varied the degrees of freedom (df) for the temperature spline between 3 and 6 to examine the impact of model flexibility on effect estimates. Third, to address potential concerns of overadjustment, we removed the seasonal sine and cosine terms and their interaction with day-of-week from the model and re-evaluated the associations. All statistical analyses were conducted using the mvmeta package in R (version 4.2.2, The R Foundation for Statistical Computing, Vienna, Austria).

## 3 Results

### 3.1 Descriptive statistics

**Figure 2:**
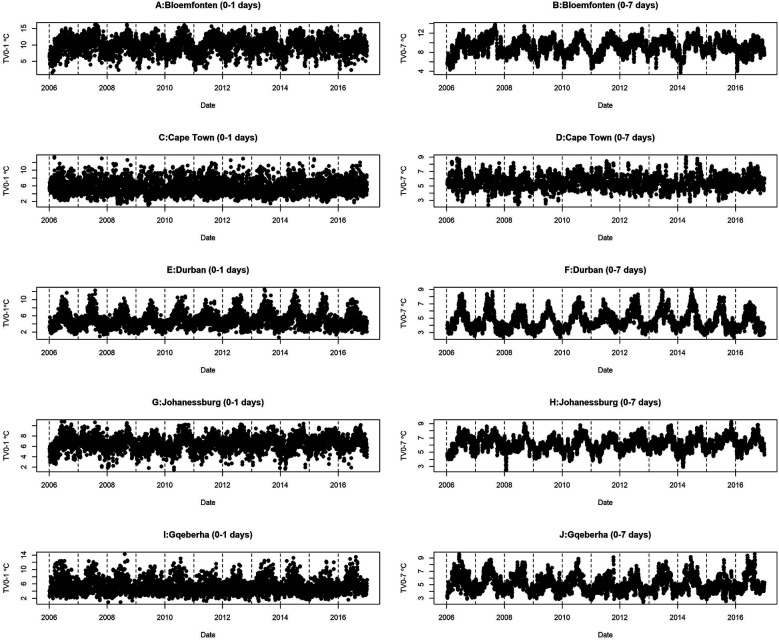
Time series distribution of TV and daily mean temperature for the five selected cities at short time lags (0–1 day) and longer time lags (0–7 days).

This study analyzed mortality and meteorological data from five South African cities between 2006 and 2016. Table 1 presents the summary statistics of respiratory and cardiovascular disease mortality counts stratified by age group and sex, alongside the mean (±SD) values of key meteorological variables across the five South African cities included in the analysis, with percentages shown in parentheses. A total of 213,875 cardiovascular disease (CVD) and 114,887 respiratory disease (RD) deaths were recorded. Durban reported the highest CVD mortality (62,623), while Bloemfontein had the lowest (15,348). For RD deaths, Johannesburg recorded the highest count (34,584), and Gqeberha the lowest (12,517). CVD mortality was higher among individuals aged ≥65 years across all cities, reaching 62.3% in Cape Town. In contrast, RD mortality was more common among those under 65 years, particularly in Durban (71.0%) and Bloemfontein (73.7%). Female mortality slightly exceeded male mortality for CVD in all cities, while RD deaths were more evenly distributed by sex. Mean temperature ranged from 16.0 ± 6.15 °C in Bloemfontein to 21.0 ± 3.19 °C in Durban. Gqeberha (Port Elizabeth) had the highest humidity (75.2 ± 9.11%), and Bloemfontein had the highest short-term temperature variability (TV0–1: 9.93 ± 2.43 °C). Figure 2 shows clear seasonal trends in daily temperature and temperature variability across all cities.

**Table 1:** Summary statistics of respiratory and cardiovascular disease mortality counts by age group and sex, as well as mean (±SD) of meteorological variables, in five South African cities. Values in parentheses represent percentages.

City	Bloemfontein	Cape Town	Durban	Johannesburg	Gqeberha
Respiratory disease					
Total	13,298	24,417	30,071	34,584	12,517
Age group					
<65	9,796 (73.7%)	12,588 (51.6%)	21,361 (71.0%)	23,489 (67.9%)	7,805 (62.4%)
≥65	3,493 (26.3%)	11,768 (48.2%)	8,604 (28.6%)	10,794 (31.2%)	4,701 (37.6%)
Sex					
Male	7,017 (52.8%)	13,450 (55.1%)	15,764 (52.4%)	18,239 (52.7%)	6,650 (53.1%)
Female	6,262 (47.1%)	10,895 (44.6%)	14,266 (47.4%)	16,093 (46.5%)	5,845 (46.7%)
Cardiovascular disease					
Total	15,348	60,654	62,623	51,653	23,597
Age group					
<65	6,899 (45.0%)	22,811 (37.6%)	27,529 (44.0%)	24,562 (47.6%)	10,408 (44.1%)
≥65	8,442 (55.0%)	37,791 (62.3%)	35,007 (55.9%)	26,907 (52.1%)	13,179 (55.9%)
Sex					
Male	7,073 (46.1%)	29,214 (48.2%)	29,278 (46.8%)	25,182 (48.8%)	10,603 (44.9%)
Female	8,260 (53.8%)	31,377 (51.7%)	33,308 (53.2%)	26,258 (50.8%)	12,977 (55.0%)
Meteorological variables (Mean ± SD)					
TV 0–1 day	9.93 ± 2.43	5.72 ± 1.91	4.88 ± 1.81	6.57 ± 1.39	5.29 ± 2.00
TV 0–2 day	9.52 ± 2.11	5.57 ± 1.56	4.75 ± 1.57	6.34 ± 1.21	5.18 ± 1.70
TV 0–3 day	9.36 ± 1.94	5.53 ± 1.36	4.71 ± 1.44	6.27 ± 1.12	5.14 ± 1.53
TV 0–4 day	9.28 ± 1.82	5.52 ± 1.22	4.69 ± 1.37	6.23 ± 1.07	5.13 ± 1.42
TV 0–5 day	9.23 ± 1.74	5.52 ± 1.11	4.68 ± 1.32	6.22 ± 1.03	5.13 ± 1.34
TV 0–6 day	9.20 ± 1.68	5.52 ± 1.03	4.67 ± 1.28	6.21 ± 1.00	5.13 ± 1.29
TV 0–7 day	9.18 ± 1.62	5.52 ± 0.971	4.67 ± 1.25	6.21 ± 0.981	5.13 ± 1.24
Temperature	16.0 ± 6.15	17.1 ± 3.85	21.0 ± 3.19	16.6 ± 4.33	17.6 ± 3.16
Humidity	53.4 ± 16.4	72.3 ± 9.95	73.8 ± 8.53	54.8 ± 19.1	75.2 ± 9.11

TV, emperature variability (from 0–1 day to 0–7 day's exposure); SD, standard deviation. Proportions in parentheses refer to percentages within each disease category (age or sex breakdowns).

### 3.2 City specific estimates

**Figure 3:**
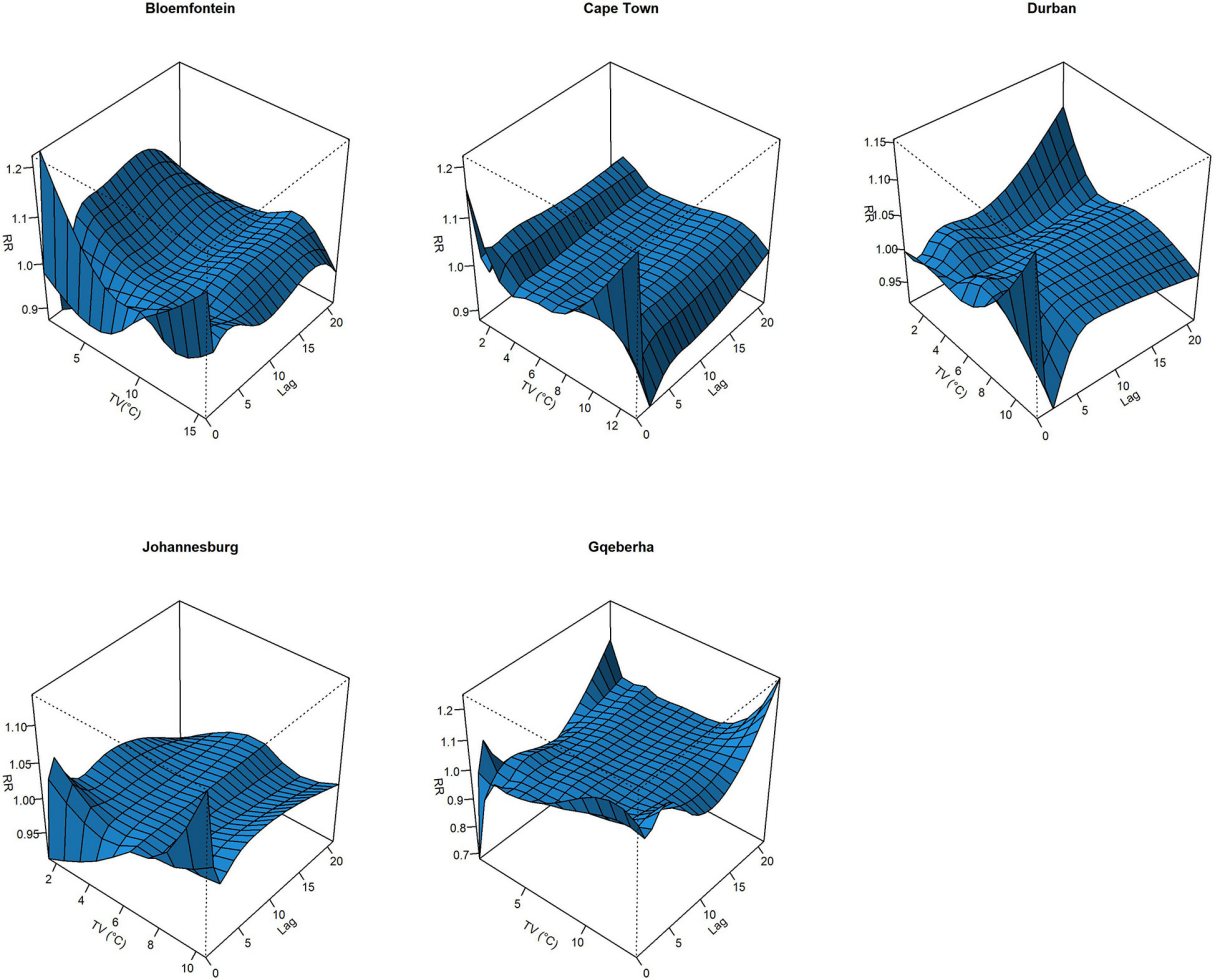
Three-dimensional graphs illustrating the exposure–lag–response relationship between short-term temperature variability (TV), based on 0–1 day exposure, and CVD mortality in five South African cities during the period 2006–2016. The *x*-axis represents TV (°C), the *y*-axis represents lag in days (up to 21), and the *z*-axis shows the estimated relative risk (RR) of CVD mortality. The 50th percentile of TV was used as the reference value.

**Figure 4:**
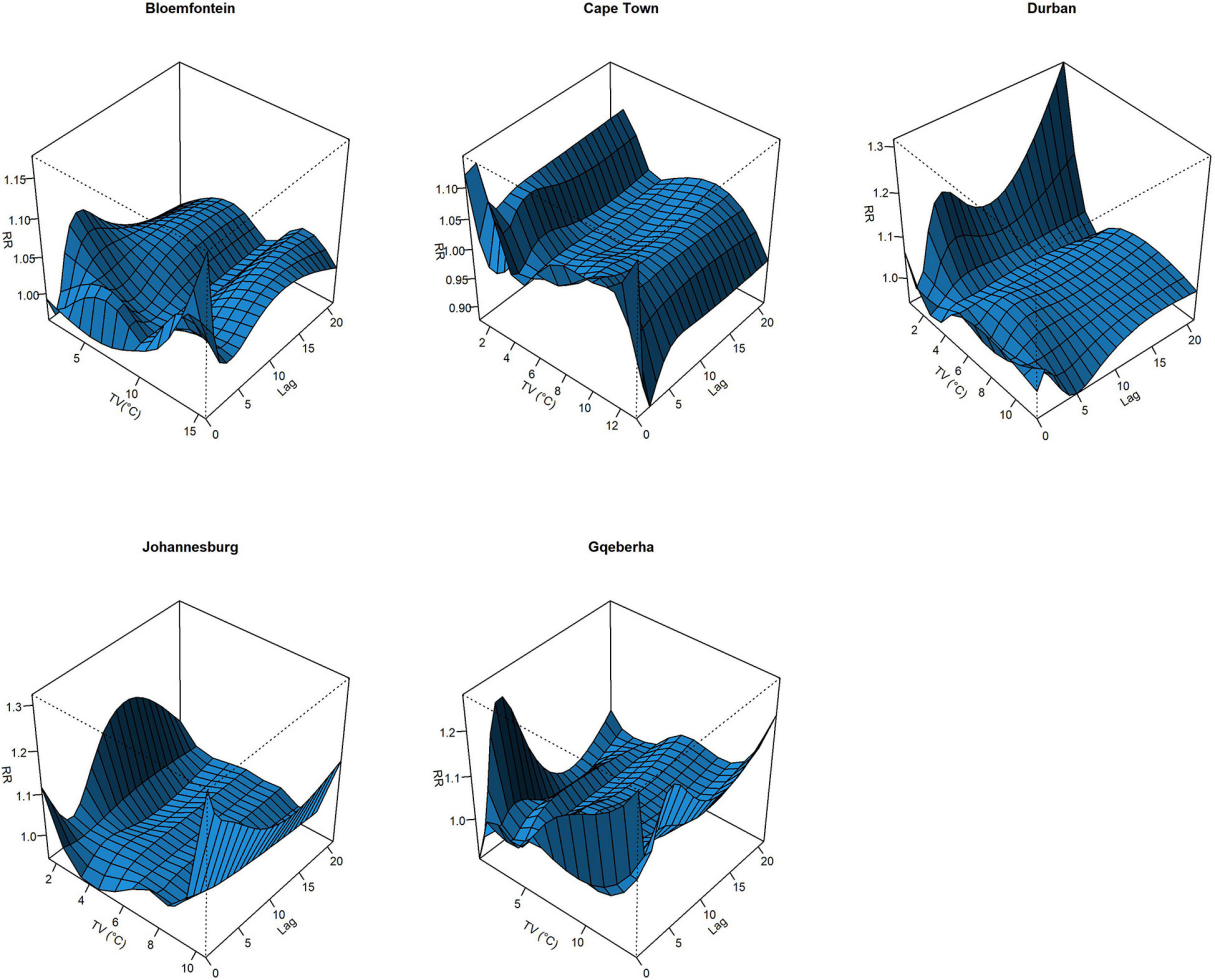
Three-dimensional graphs illustrating the exposure–lag–response relationship between short-term temperature variability (TV), based on 0–1 day exposure, and RD mortality in five South African cities during the period 2006–2016. The *x*-axis represents TV (°C), the *y*-axis represents lag in days (up to 21), and the *z*-axis shows the estimated relative risk (RR) of RD mortality. The 50th percentile of TV was used as the reference value.

**Figure 5:**
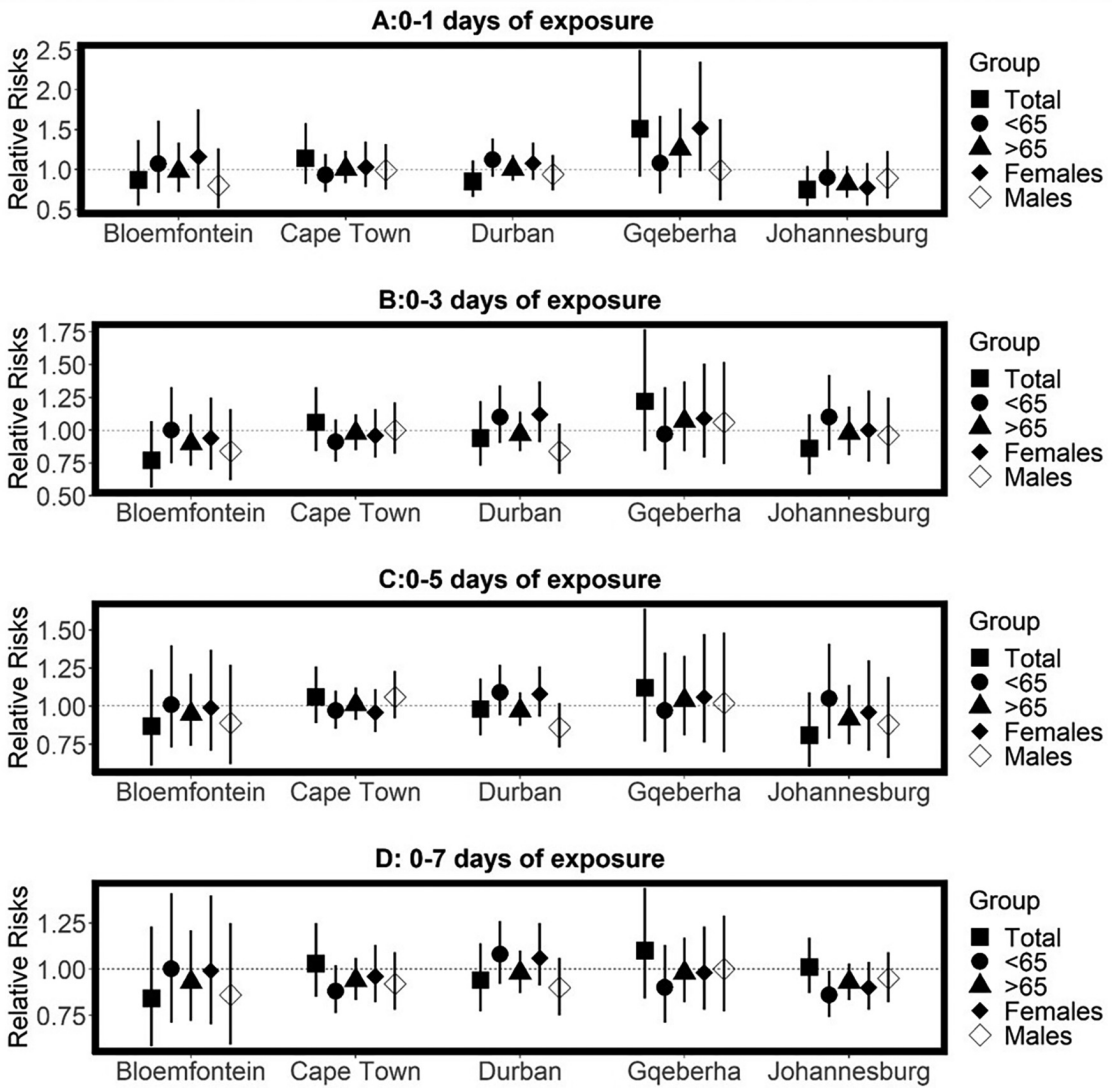
Estimated effects of temperature variability (TV) on cardiovascular disease (CVD) mortality by age group, sex, and overall population at different lag periods: **(A)** 0–1 day, **(B)** 0–3 days, **(C)** 0–5 days, and **(D)** 0–7 days of exposure. Relative risks (RRs) and 95% confidence intervals (CIs) are presented for each city, comparing the 99th percentile of TV to the 50th percentile (reference level). Estimates are stratified by age.

**Figure 6:**
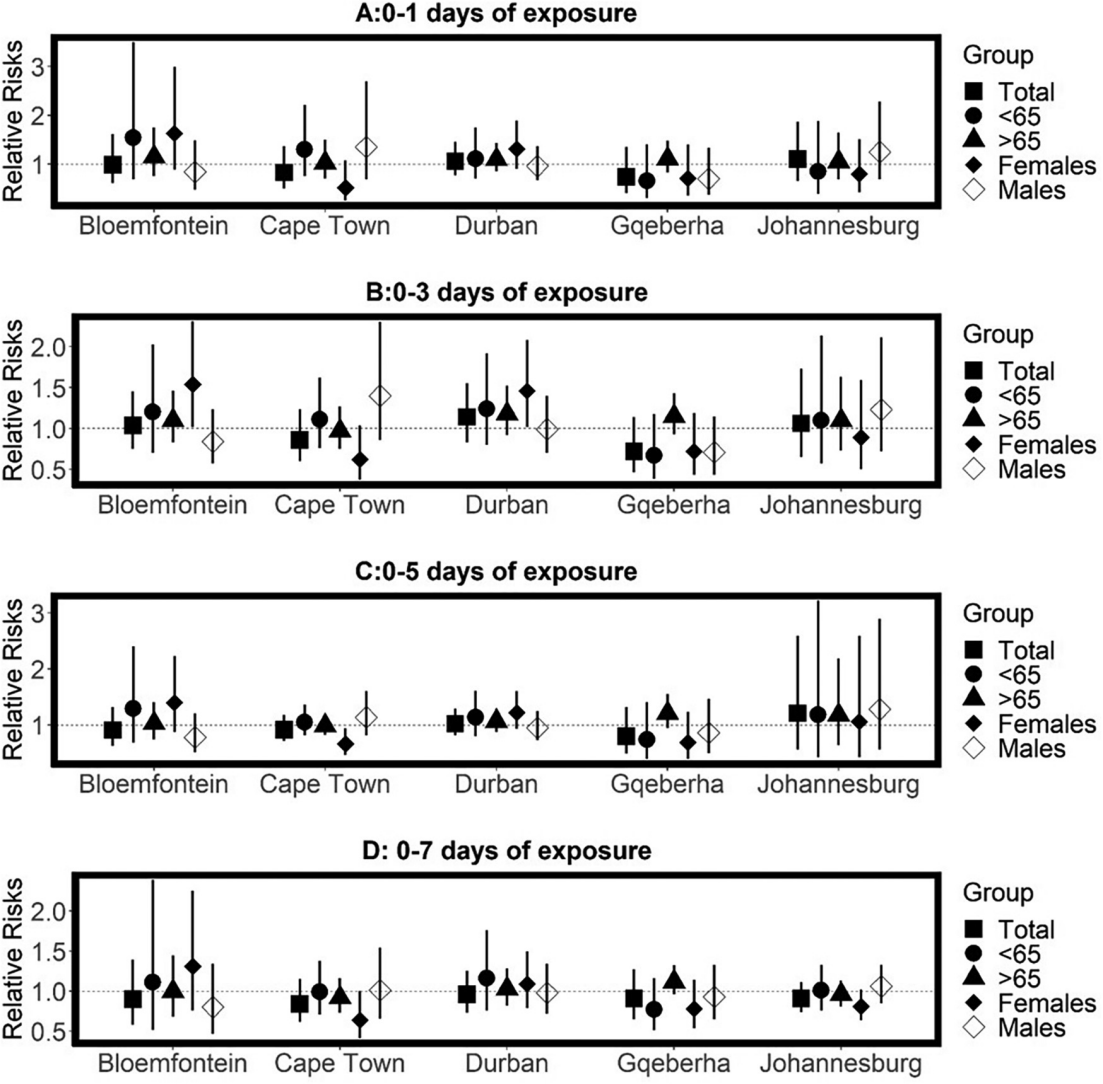
Estimated effects of temperature variability (TV) on respiratory disease mortality by age group, sex, and overall population at different lag periods: **(A)** 0–1 day, **(B)** 0–3 days, **(C)** 0–5 days, and **(D)** 0–7 days of exposure. Relative risks (RRs) and 95% confidence intervals (CIs) are presented for each city, comparing the 99th percentile of TV to the 50th percentile (reference level). Estimates are stratified by age.

The complex associations between CVD and RD mortality and TV (based on 0–1 days TV exposure) are illustrated in the three-dimensional plots shown in Figures 3, 4. The 50th percentile of TV level was applied as the reference level. The mortality risks associated with an increase in TV (for different exposure days) from the 50th to the 99th percentile, after adjusting for the confounders, are shown in Figures 5, 6. In general, greater CVD effects appeared at short exposure durations (0–1 day). The elderly and females were more vulnerable to die from CVD in most of the cities, except for Cape Town and Gqeberha. Delayed CVD risks were observed for males in Johannesburg. CVD risks were higher in Gqeberha compared to the other cities. In general, there were positive associations between TV and RD mortality in all the cities and the effects appeared to last until 0–7 days exposure durations. The elderly were more vulnerable to die from RD in Bloemfontein, Cape Town, and Johannesburg. Females were more vulnerable to RD deaths in Bloemfontein and Durban, while males were more vulnerable to RD deaths in Cape Town and Johannesburg. RD risks did not differ by age or sex in Gqeberha, while RD risks were generally higher in Bloemfontein and Johannesburg as compared to the other cities.

### 3.3 Meta-analysis

The pooled results across the five cities suggested no statistically significant TV effect on CVD mortality. However, in general, the elderly and females were exposed to high CVD mortality risks with increasing TV (Supplementary Table A3). Table 2 presents the pooled relative risks (RRs) and 95% confidence intervals (CIs) for RD mortality associated with temperature TV across different exposure windows (0–1 to 0–7 days), stratified by age group (<65 and ≥65 years) and sex (females and males). In general, positive, and immediate RD mortality risks associated with TV at different reference points (25th, 75th and 99th percentiles) were observed for all ages combined, and the risks remained positive for up to 0–7 days. An increase in RD mortality risks (RR = 1.21 95% CI: 1.04, 1.38) per TV (at 0–2 days exposure duration) increase from the 25th to 50th percentile observed for all ages combined. After stratification by age, statistically significant RD mortality risks per TV increase from the 50th to the 75th percentile were observed for the elderly at 0–2 days exposure duration (RR = 1.18, 95% CI: 1.04, 1.32), at 0–3 days0–2 days exposure duration (RR = 1.16, 95% CI: 1.04, 1.28) and 0–7 days 0–2 days exposure duration (RR = 1.12, 95% CI: 1.02, 1.22). In general, the elderly were exposed to a higher RD mortality risk, compared to the adults (<65-years). Stratification by sex showed that the RD mortality risks differed by sex, refence points, and lags. For example, higher risks of RD mortality per TV increase from the 50th to the 75th percentile were observed at short exposure durations (0–1 days) for females (RR = 1.18 95% CI: 0.85, 1.50) while protective effects were observed for males (RR = 0.99, 95% CI: 0.85;1.13). In general, RD mortality risks were higher for males at long exposure durations and females had higher risk at short exposure durations.

**Table 2:** Meta-analysis of the relative risks of the association between RD mortality and TV. Models were adjusted for day of the week, public holiday, time, daily mean temperature and relative humidity.

Exposure	Percentiles	Respiratory mortality				
		All ages	<65	≥65	Females	Males
TV_0−1_	Low^a^	1.04 (0.90;1.18)	1.06 (0.86;1.27)	0.91 (0.77;1.05)	0.90 (0.78;1.02)	0.96 (0.83;1.08)
	High^b^	0.99 (0.85;1.13)	1.11 (0.84;1.38)	1.04 (0.89;1.19)	1.18 (0.85;1.50)^d^	0.99 (0.85;1.13)
	Extreme high^c^	0.99 (0.76;1.23)	0.98 (0.78;1.18)	1.12 (0.84;1.39)	1.04 (0.65;1.43)^d^	0.99 (0.76;1.23)
TV_0−2_	Low^a^	1.21 (1.04;1.38)*	1.22 (0.94;1.50)	0.99 (0.77;1.21)	0.87 (0.64;1.09)	1.18 (0.96;1.39)
	High^b^	1.05 (0.94;1.17)	1.03 (0.91;1.16)	1.18 (1.04;1.32)*	1.00 (0.88;1.13)	1.05 (0.94;1.17)
	Extreme high^c^	1.04 (0.85;1.22)	0.95 (0.78;1.11)	1.12 (0.91;1.35)	0.90 (0.70;1.25)	1.04 (0.85;1.22)
TV_0−3_	Low^a^	1.18 (0.92;1.45)^d^	1.05 (0.84;1.25)	1.02 (0.71;1.34)	0.91 (0.66;1.55)	1.17 (0.93;1.41)
	High^b^	1.03 (0.92;1.13)	1.04 (0.90;1.19)	1.16 (1.04;1.28)*	0.98 (0.86;1.10)	1.03 (0.92;1.13)
	Extreme high^c^	1.01 (0.79;1.22)	0.99 (0.82;1.15)	1.09 (0.88;1.31)	1.08 (0.69;1.46)^d^	1.01 (0.79;1.22)
TV_0−4_	Low^a^	1.13 (0.94;1.33)	1.00 (0.80;1.20)	0.98 (0.68;1.27)	0.92 (0.68;1.16)	1.07 (0.84;1.29)
	High^b^	1.00 (0.90;1.09)	1.00 (0.93;1.08)	1.07 (0.97;1.17)	1.01 (0.91;1.11)	1.00 (0.90;1.09)
	Extreme high^c^	0.95 (0.77;1.33)	0.96 (0.80;1.12)	1.07 (0.85;1.29)	1.02 (0.69;1.34)^d^	0.95 (0.77;1.13)
TV_0−5_	Low^a^	1.12 (0.99;1.25)	1.05 (0.85;1.24)	1.10 (0.80;1.40)	0.98 (0.75;1.21)	1.14 (0.92;1.36)
	High^b^	1.00 (0.91;1.10)	1.03 (0.91;1.15)	1.09 (0.98;1.19)	1.05 (0.93;1.16)	1.00 (0.91;1.10)
	Extreme high^c^	0.98 (0.80;1.15)	0.96 (0.81;1.10)	1.07 (0.89;1.26)	1.02 (0.70;1.33)^d^	0.98 (0.80;1.15)
TV_0−6_	Low^a^	1.06 (0.93;1.20)	1.03 (0.83;1.22)	1.07 (0.77;1.36)	0.96 (0.72;1.20)	1.11 (0.89;1.34)
	High^b^	1.01 (0.93;1.09)	1.04 (0.94;1.14)	1.05 (0.96;1.14)	1.01 (0.92;1.09)	1.01 (0.93;1.09)
	Extreme high^c^	1.02 (0.87;1.16)	0.93 (0.81;1.05)	1.07 (0.91;1.24)	0.96 (0.66,1.27)^d^	1.02 (0.87;1.16)
TV_0−7_	Low^a^	1.05 (0.92;1.18)	0.99 (0.79;1.79)	1.02 (0.73;1.32)	0.93 (0.70;1.17)	1.07 (0.85,1.29)
	High^b^	1.05 (0.97;1.14)	1.09 (1.02;1.17)*	1.12 (1.02;1.22)*	1.10 (0.95;1.25)^d^	1.05 (0.97;1.14)
	Extreme high^c^	1.00 (0.85;1.14)	0.91 (0.78;1.03)	0.99 (0.83;1.16)	0.90 (0.72;1.07)	1.00 (0.85;1.14)

TV-temperature variability at different exposure windows (0–1 day to 0–7 days).

^a^ Low TV exposure = 25th percentile compared to the reference level at the 50th percentile.

^b^ High TV exposure = 75th percentile compared to the reference level at the 50th percentile.

^c^ Extreme high TV exposure = 99th percentile compared to the reference level at the 50th percentile.

^d^ Heterogeneity among the cities was suggested by a *p*-value < 0.1 from the test for heterogeneity. These cities were analyzed by random effects.

* Statistically significant *p*-value < 0.05.

## 4 Discussion

In this study distributed lag non-linear models, combined with quasi-Poisson generalized linear models were used to examine the exposure-response association and delayed effects between TV and mortality in five South African cities, located in different Köppen-Geiger climatic zones. The relationship between TV and mortality was found to be non-linear, deviating from the linear assumptions employed in earlier studies conducted in various settings such as China, Europe, and the United States (10, 23). Although studies utilizing intraday (e.g., diurnal temperature range) and interday variability have also applied non-linear models (19, 24, 25), our findings reinforce the importance of applying flexible methods such as DLNM, particularly when exploring multiple lag structures and complex exposure–response curves. In line with prior studies, the effects of TV appeared to last up to 0–7 days after exposure (10, 26, 27), which emphasizes the need to assess several lengths of TV and lags. In this study, the mortality risks did not increase with exposure days. Similar to Tian et al. (27), the highest risks were observed at 0–1 day of exposure. The elevated risks observed at short durations (lags) in this study, may also reflect mortality displacement (harvesting), where vulnerable individuals such as the elderly or chronically ill die slightly earlier due to stress induced by temperature variability. This phenomenon has been documented in prior heat-related mortality studies and should be considered when interpreting the transient nature of the effects (28).

The mortality risks varied across the five cities, with high risks observed in inland cities, especially in Bloemfontein, which denotes a semi-arid region. Similar to findings from a previous study conducted in the semi-arid regions of China, females in Bloemfontein exhibited a higher vulnerability to respiratory disease (RD) mortality associated with temperature variation (29). Inland cities tend to experience high TV due to physiographic factors, such as lack of oceans or seas, which act as moderating influences on temperature (30). People might experience discomfort due to abrupt fluctuations in temperature throughout the day and between days, primarily because they lack adequate physiological and behavioral adaptations to cope with such changes (31). Individuals in coastal areas were more vulnerable to mortality following prolonged exposure to TV, such as Durban which experiences higher average temperatures, in contrast to the findings reported by Guo et al. (10) where acute mortality risks were observed in warm areas. Further research is needed to better understand the differential impacts of temperature variability on mortality between inland and coastal areas.

Consistent with global studies (23, 32), RD mortality risks associated with TV were generally higher than those for CVD. However, the results contradict the results of a recent study conducted in Cape Town which found higher risks of CVD hospital admissions as compared to RD admissions (15). The observed differences may be attributed to the demographic characteristics of the study populations and the nature of health outcomes assessed. For instance, the recent study focused on hospital admissions among private hospital patients, whereas the present study assessed mortality outcomes in the general population. Hospital admission data may capture less severe cases or a subset of the population with better healthcare access, while mortality data reflects more severe health outcomes. Furthermore, although several TV exposure durations were assessed in both studies, different modeling approaches were used. The underlying mechanisms explain why increased temperature variability may lead to elevated mortality risks, particularly among certain vulnerable subgroups that remain poorly understood and warrant further investigation in future research.

The elderly population were likely more susceptible to TV, which is similar to findings from previous studies. This might be due to the degenerating thermoregulatory system linked with ageing (10, 27, 33), but also to the higher prevalence of chronic conditions, such as diabetes, cardiovascular disease, and hypertension, which can amplify physiological stress during temperature fluctuations. These underlying conditions may also interact with TV in younger age groups living with comorbidities, although this requires further investigation. Furthermore, subgroup analysis showed that females were generally more affected by TV, which is consistent with previous studies (34, 35). Females are often considered more vulnerable to temperature changes due to a combination of physiological and societal factors. Physiologically, hormonal fluctuations related to the menstrual cycle, particularly the effects of estrogen and progesterone can influence core body temperature, vasodilation, and sweating responses, potentially altering thermoregulation and increasing susceptibility to environmental stressors (36). Socially, gender-based differences in occupational exposure, clothing norms, caregiving responsibilities, and healthcare access may exacerbate this vulnerability, particularly in low- and middle-income countries where women often face structural barriers to health services and adaptive capacity (2, 5). Together, these factors may increase the risk of temperature-related adverse outcomes in females, especially in socioeconomically and climatically vulnerable settings. In addition, hormones, such as estrogen, can influence how the body perceives temperature. Fluctuations in estrogen levels during the menstrual cycle may contribute to varying temperature sensitivity in women (35). Furthermore, occupational exposures are also likely to influence vulnerability to temperature variability, with outdoor workers potentially at greater risk (5). Also, adaptation strategies like improved housing, access to cooling or heating, and early warning systems can reduce the health impacts of temperature variability (4, 5), especially in vulnerable communities. Furthermore, behavioral practices such as hydration, or clothing, which may influence individual vulnerability to temperature variability. Future research should consider these factors to better understand how people adapt to fluctuating temperatures. These factors are important modifiers of heat-related health risks (5) and should be considered in future research.

This study has some specific strengths. Firstly, this is the first multi-city study focusing on the relationship between TV and mortality in South Africa, using a composite index of intra- and interday variability (10). Secondly, most of the studies that used the compositive index of TV assumed the association between TV and mortality to be linear (10, 18, 19). In the present study, the non-linear and delayed effects of TV at different exposure days were investigated. Thirdly, cause-specific, age-specific, and sex-specific mortality was investigated. Fourthly, to test the robustness of the results, a range of sensitivity analyses were performed (Supplementary Figures A1–A10 in the appendix).

This study also has some limitations. Firstly, this study was conducted at the population level using city-wide average meteorological data. As such, exposure misclassification may be a source of bias, since temperature and humidity can vary within cities due to local environmental conditions, urban heat islands, and individual behaviors. Secondly, the cities included in the study are in urban areas and therefore, the results might not be generalizable to rural areas. Even within urban settings, people's living conditions, access to healthcare, and types of work can affect how temperature variability impacts health. These differences matter, and future studies should consider including socio-economic factors and rural areas to better understand who is most at risk. Remote sensing data, such as satellite-derived temperature, could improve spatial resolution by capturing local microclimates, especially between urban and rural areas. Though not used here, it may benefit future studies. However, the cities were in different Köppen-Geiger climatic zones. Thus, the results can be generalized to areas with similar climatic conditions. Thirdly, air pollutants which are known to confound heat and health associations were not controlled due to the availability of adequate data. However, previous studies found little to no effect of air pollutants on the overall results (15, 37). Fourthly, stratification by season was not performed. Lastly, while this study focused specifically on temperature variability as an exposure, it is important to distinguish TV from other climate-related stressors such as heatwaves and cold spells. Heatwaves and cold spells are acute extreme events that tend to cause short-term spikes in mortality, whereas TV reflects frequent and irregular fluctuations in temperature that can lead to more sustained physiological stress. Prior studies have shown that both types of exposure are associated with increased health risks, but through potentially different mechanisms and time frames (38, 39). Future research should consider comparing these exposures within the same analytical framework to better understand their relative and combined impacts on mortality.

Given the increasing global frequency and intensity of temperature extremes, as recently projected by García-León et al. (39, 40), urgent integration of environmental risk factors like TV into epidemiological surveillance and public health strategies is essential, especially in vulnerable regions like southern Africa (3). Future research should further standardize and compare different TV metrics to isolate the most predictive measures and inform targeted public health interventions.

## 5 Conclusion

This study advances the knowledge of mortality risk factors in South Africa. The study found a positive association between TV and mortality. The increased risks of death due to TV highlight the importance of assessing the health effects of other indicators of climate change, and not only focusing on mean temperature. However, little to no statistically significant effects of TV on CVD mortality were observed, with the elderly and females more vulnerable. Significant RD mortality risks were observed at the national level. Furthermore, the elderly were more vulnerable to RD mortality; in terms of sex in general, females were more vulnerable. Further research can provide a more comprehensive understanding of the mortality risks associated with TV exposure, allowing for more targeted and evidence-based policies and interventions. This study could help policymakers and clinicians to inform public health strategies and clinical practices aimed at reducing mortality rates and improving the overall health of the population, especially among vulnerable groups, like the elderly and females.

## Data Availability

The original contributions presented in the study are included in the article/Supplementary Material, further inquiries can be directed to the corresponding author.
