# Supplementary material for: Examination of the association between temperature variability and cardiovascular and respiratory mortality in South Africa, 2006–2016
Source: Front Epidemiol. 2025 Jun 3;5:1553553. doi: 10.3389/fepid.2025.1553553 (PMC12172194; doi:10.3389/fepid.2025.1553553)
Supplement: Supplementary file 1 [file Supplementaryfile1.docx]

Supplementary Material

# Supplementary Tables

**Table A 1.** Specifications of the cross-basis function of TV by city when the outcome was CVD mortality

| **City** | **TV** | **Lag structure** |
| --- | --- | --- |
| **Bloemfontein** | $list (fun="ns", df=4)$ | $arglag=(fun="bs",degree=3, knots=lk)$ |
| **Cape Town** | $list (fun="ns", df=4)$ | $arglag=(fun="bs",degree=3, knots=lk)$ |
| **Durban** | $list (fun="ns", df=4)$ | $arglag=(fun="ns",df=4, knots=lk)$ |
| **Johannesburg** | $list (fun="ns", df=4)$ | $arglag=(fun="ns",df=4, knots=lk)$ |
| **Gqeberha** | $list (fun="ns", df=4)$ | $arglag=(fun="ns",df=4, knots=lk)$ |
| $ns-$natural cubic spline with four degrees of freedom  $bs$К – B-spline with three degrees of freedom  Lk – quantile (TV01, c (.25, .50, .75), na.rm = T) | | |

**Table A 2.** Specifications of the cross-basis function of TV by city when the outcome was RD mortality

| City | TV | Lag structure |
| --- | --- | --- |
| Bloemfontein | $list (fun="ns", df=4)$ | $arglag=(fun="bs",degree=3, knots=lk)$ |
| Cape Town | $list (fun="ns", df=4)$ | $arglag=(fun="bs",degree=3, knots=lk)$ |
| Durban | $list (fun="ns", df=4)$ | $arglag=(fun="ns",df=4, knots=lk)$ |
| Johannesburg | $list (fun="ns", df=4)$ | $arglag=(fun="bs",degree=3, knots=lk)$ |
| Gqeberha | $list (fun="ns", df=4)$ | $arglag=(fun="ns",df=4, knots=lk)$ |
| $ns-$natural cubic spline with four degrees of freedom  $df$ =degrees of freedom  $bs$- B-spline with three degrees of freedom  lk- quantile (TV01, c (.25, .50, .75), na.rm = T) | | |

**Table A3.** Meta-analysis of the relative risks of the association between Cardiovascular mortality and TV. Models were adjusted for day of the week, public holiday, time, daily mean temperature and relative humidity

| **Exposure Variable** |  | **Cardiovascular mortality** | | | | |
| --- | --- | --- | --- | --- | --- | --- |
|  |  | **All ages** | **<65** | **≥65** | **Females** | **Males** |
| TV_0-1_ | Low ^a^ | 0.98 (0.94; 1.02) | 0.95 (0.89; 1.01) | 0.98 (0.93; 1.04) | 0.95 (0.9; 1.01) | 1.02 (0.96; 1.08) |
|  | High ^b^ | 0.94 (0.89; 0.99) | 0.88 (0.79; 0.97) | 0.94 (0.86; 1.03) | 0.93 (0.84; 1.02) | 0.93 (0.85; 1) |
|  | Extreme high ^c^ | 1 (0.9; 1.1) | 0.98 (0.76; 1.21) | 1.02 (0.89; 1.15) | 1.08 (0.89; 1.27) | 0.93 (0.79; 1.08) |
| TV_0-2_ | Low ^a^ | 0.92 (0.67; 1.17) | 0.92 (0.62; 1.21) | 0.95 (0.79; 1.11) | 0.97 (0.71; 1.24) | 0.89 (0.65; 1.13) |
|  | High ^b^ | 0.96 (0.92; 1) | 0.99 (0.91; 1.07) | 0.94 (0.88; 1) | 0.98 (0.92; 1.03) | 0.93 (0.86; 1) |
|  | Extreme high ^c^ | 0.96 (0.88; 1.04) | 0.96 (0.83; 1.08) | 0.98 (0.88; 1.09) | 1.01 (0.89; 1.13) | 0.92 (0.81; 1.03) |
| TV_0-3_ | Low ^a^ | 0.97 (0.8; 1.14) | 0.87 (0.68; 1.07) | 1.05 (0.88; 1.22) | 1.08 (0.68; 1.47) | 0.89 (0.69; 1.08) |
|  | High ^b^ | 0.97 (0.93; 1.02) | 0.98 (0.92; 1.05) | 0.99 (0.93; 1.05) | 1.03 (0.97; 1.09) | 0.92 (0.86; 0.98) |
|  | Extreme high ^c^ | 0.98 (0.9; 1.06) | 0.96 (0.84; 1.09) | 1.01 (0.91; 1.11) | 1.02 (0.91; 1.13) | 0.94 (0.82; 1.05) |
| TV_0-4_ | Low ^a^ | 0.95 (0.8; 1.11) | 0.88 (0.7; 1.07) | 1.01 (0.85; 1.17) | 1.03 (0.73; 1.32) | 0.89 (0.71; 1.07) |
|  | High ^b^ | 0.98 (0.94; 1.02) | 1.01 (0.94; 1.08) | 0.97 (0.92; 1.02) | 1.01 (0.96; 1.06) | 0.95 (0.9; 1.01) |
|  | Extreme high ^c^ | 0.93 (0.84; 1.01) | 0.99 (0.85; 1.12) | 0.97 (0.86; 1.07) | 0.98 (0.87; 1.09) | 0.87 (0.75; 0.99) |
| TV_0-5_ | Low ^a^ | 0.97 (0.88; 1.05) | 0.87 (0.74; 1) | 1.05 (0.93; 1.16) | 0.97 (0.85; 1.08) | 0.96 (0.84; 1.09) |
|  | High ^b^ | 1 (0.96; 1.03) | 1.02 (0.97; 1.08) | 1 (0.95; 1.04) | 1 (0.95; 1.05) | 0.99 (0.93; 1.05) |
|  | Extreme high ^c^ | 0.99 (0.92; 1.05) | 0.99 (0.88; 1.1) | 1.02 (0.93; 1.11) | 1.01 (0.92; 1.1) | 0.95 (0.84; 1.07) |
| TV_0-6_ | Low ^a^ | 0.96 (0.88; 1.04) | 0.86 (0.73; 0.99) | 1.05 (0.94; 1.16) | 0.95 (0.84; 1.07) | 0.96 (0.83; 1.09) |
|  | High ^b^ | 0.99 (0.96; 1.02) | 1.03 (0.98; 1.07) | 0.99 (0.95; 1.04) | 0.98 (0.94; 1.02) | 1.01 (0.96; 1.06) |
|  | Extreme high ^c^ | 0.98 (0.92; 1.04) | 1.01 (0.92; 1.1) | 0.98 (0.9; 1.05) | 0.99 (0.91; 1.07) | 0.98 (0.89; 1.06) |
| TV_0-7_ | Low ^a^ | 0.95 (0.87; 1.03) | 0.87 (0.74; 0.99) | 1.03 (0.92; 1.14) | 0.96 (0.84; 1.07) | 0.95 (0.83; 1.07) |
|  | High ^b^ | 1 (0.97; 1.03) | 1.03 (0.98; 1.07) | 1.01 (0.98; 1.05) | 1 (0.96; 1.04) | 1.01 (0.96; 1.06) |
|  | Extreme high ^c^ | 0.95 (0.89; 1.01) | 1 (0.9; 1.09) | 0.94 (0.84; 1.04) | 0.98 (0.89; 1.06) | 0.93 (0.85; 1.02) |
| TV-temperature variability, ^a^ Low TV exposure = 25th percentile compared to the reference level at the 50th percentile. ^b^ High TV exposure = 75th percentile compared to the reference level at the 50th percentile Extreme high TV exposure = 99th percentile compared to the reference level at the 50th percentile., ^d^ Heterogeneity among the cities was suggested by a p-value < 0.1 from the test for heterogeneity. These cities were analyzed by random effects. *Statistically significant p-value<0.05 | | | | | | |


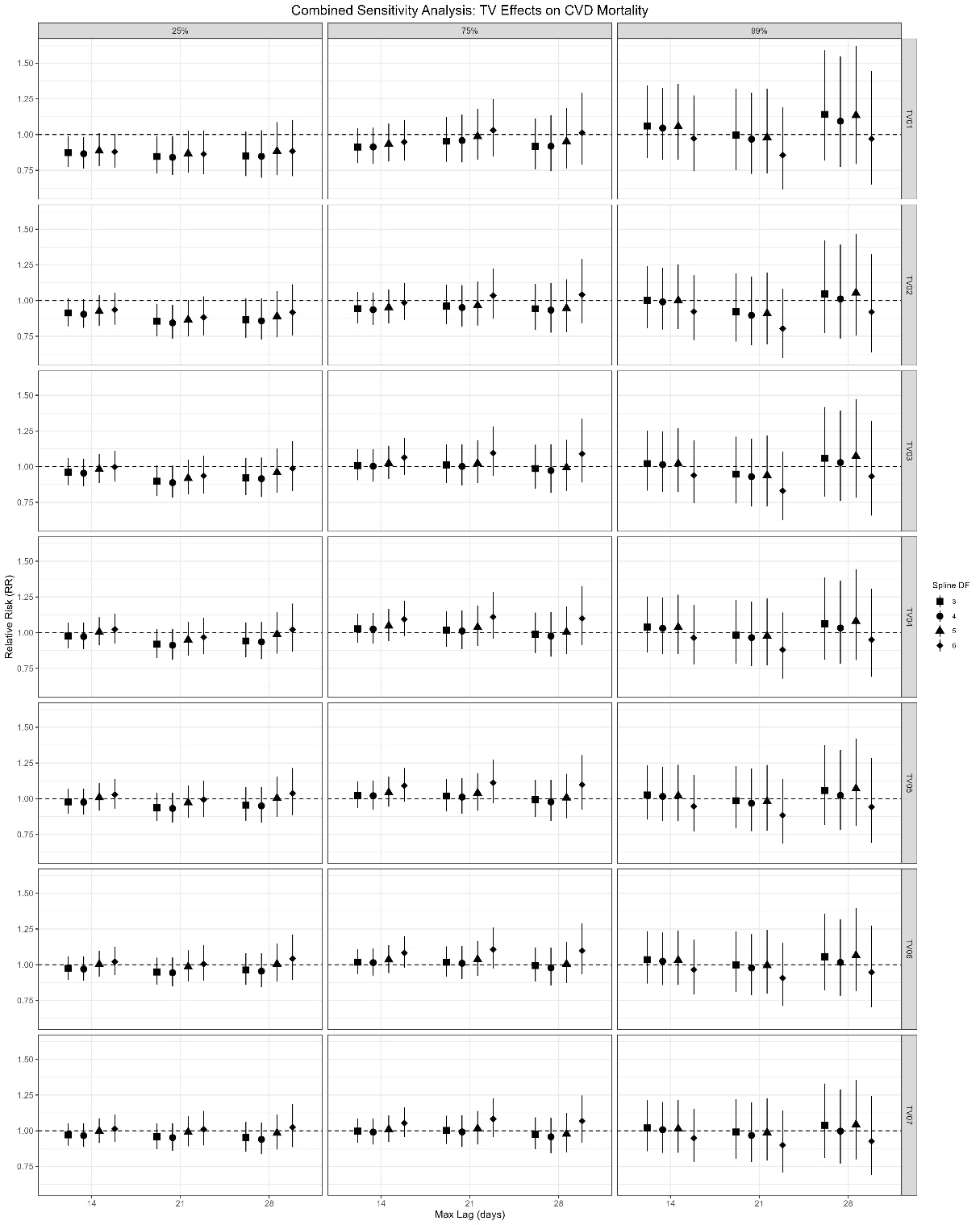


Figure A1. Sensitivity analysis of temperature variability (TV01–TV07) effects on cardiovascular mortality in Bloemfontein at the 25th, 75th, and 99th percentiles. Points indicate relative risks (RR), vertical lines are 95% confidence intervals, and the dashed line represents no effect (RR = 1). Analysis varied spline degrees of freedom (3–6) and lag durations (14,21,28 days).


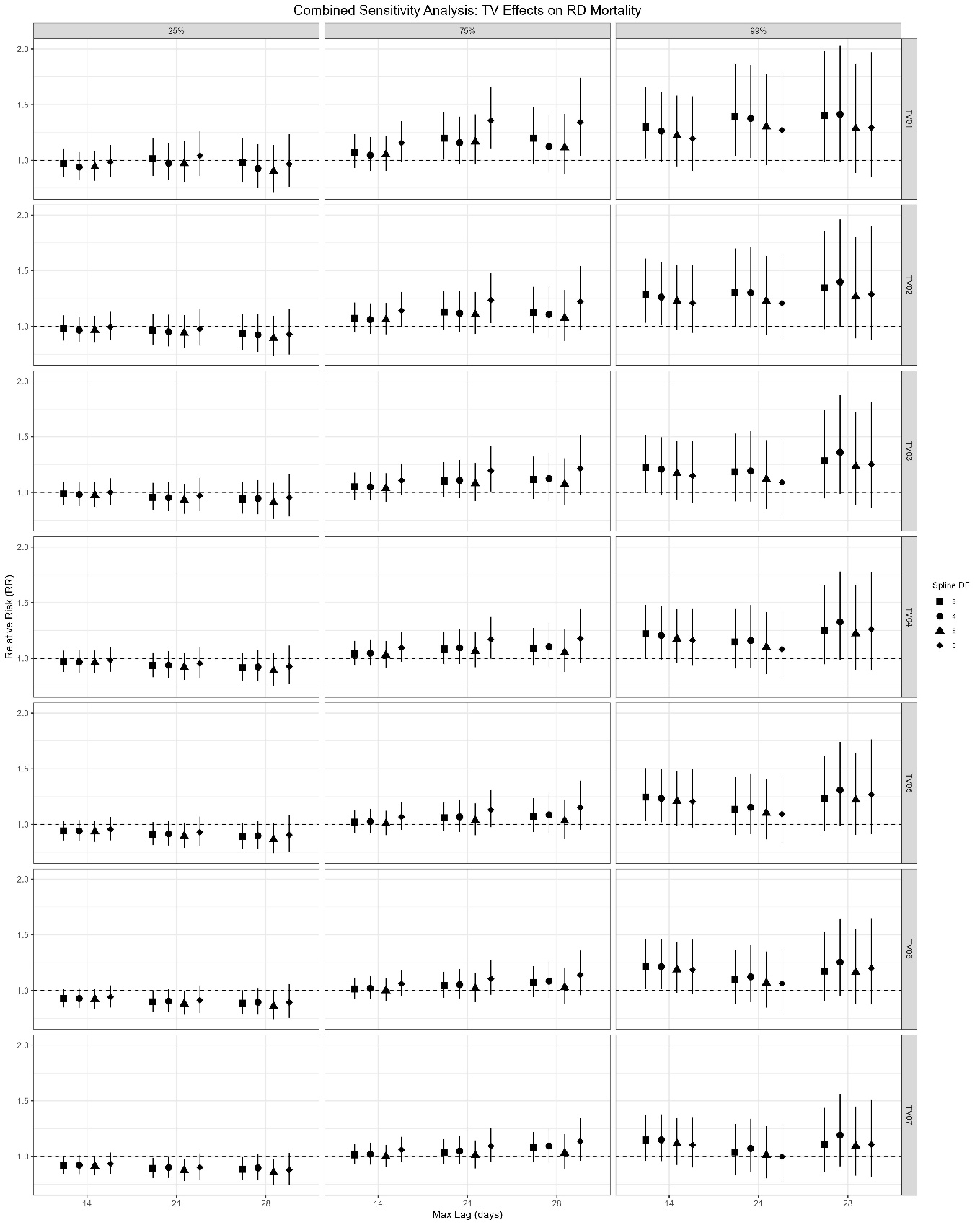


Figure A2. Figure A1. Sensitivity analysis of temperature variability (TV01–TV07) effects on respiratory mortality in Bloemfontein at the 25th, 75th, and 99th percentiles. Points indicate relative risks (RR), vertical lines are 95% confidence intervals, and the dashed line represents no effect (RR = 1). Analysis varied spline degrees of freedom (3–6) and lag durations (14,21,28 days).


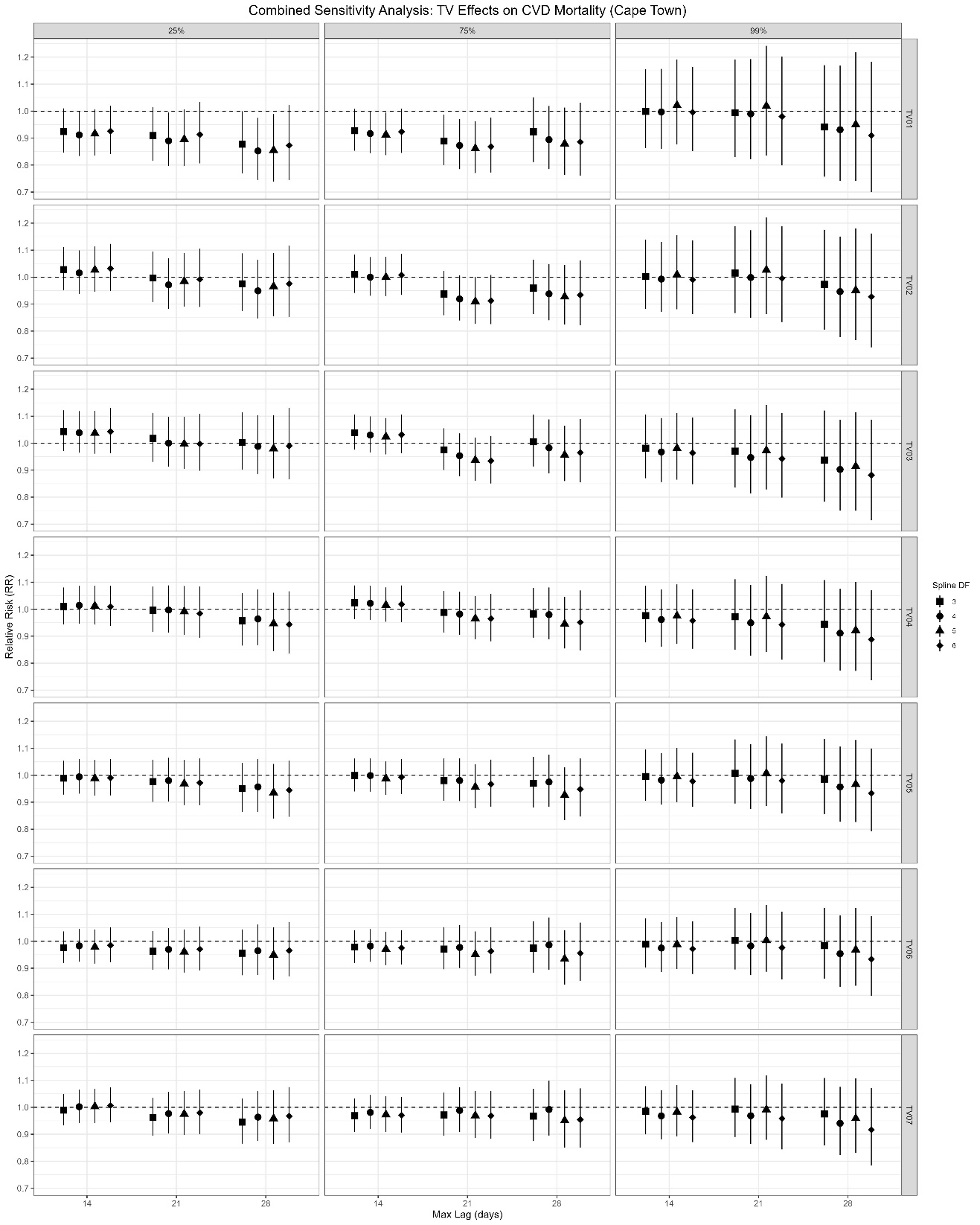


Figure A3. Sensitivity analysis of temperature variability (TV01–TV07) effects on cardiovascular mortality in Cape Town at the 25th, 75th, and 99th percentiles. Points indicate relative risks (RR), vertical lines are 95% confidence intervals, and the dashed line represents no effect (RR = 1). Analysis varied spline degrees of freedom (3–6) and lag durations (14,21,28 days).


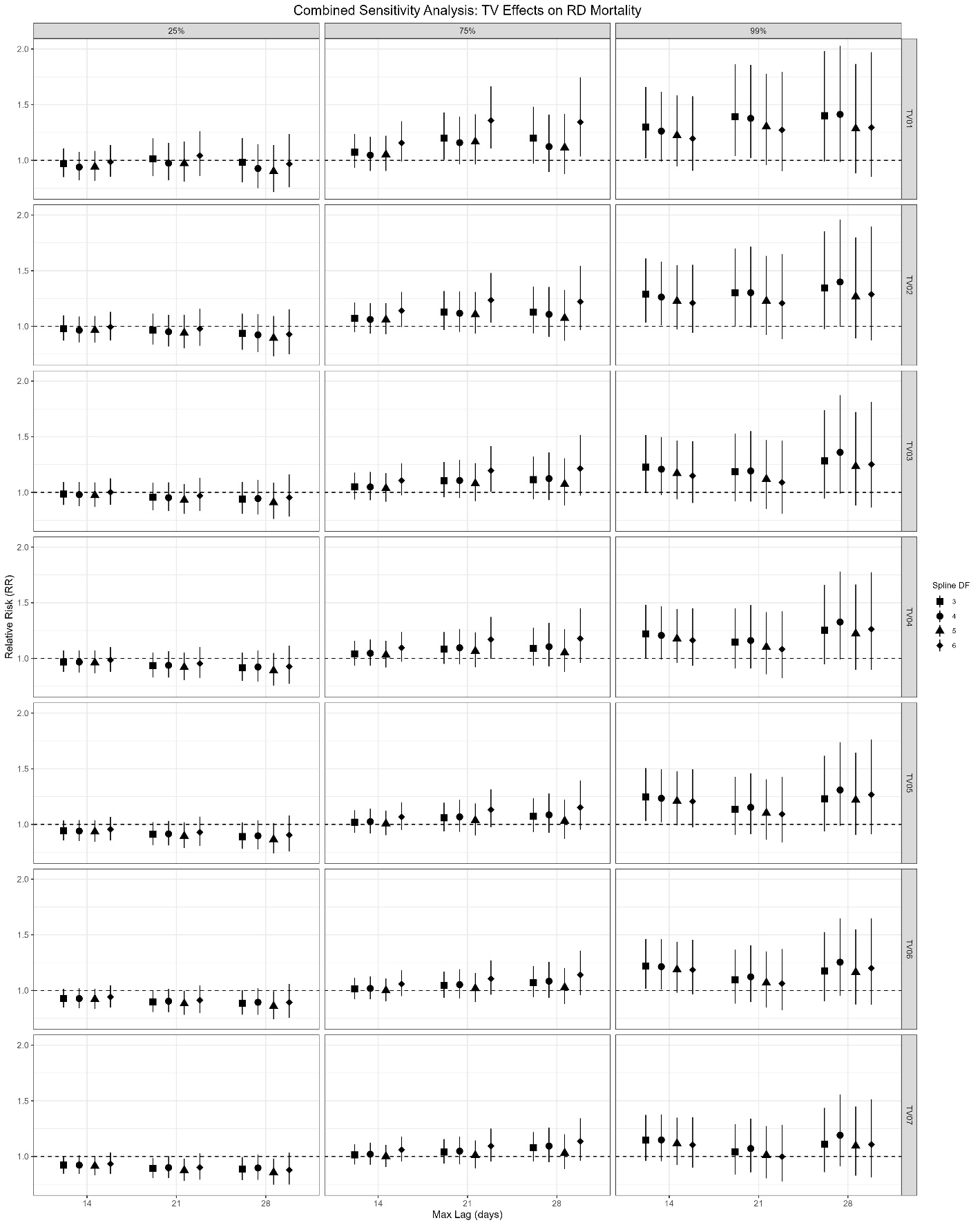


Figure A4. Sensitivity analysis of temperature variability (TV01–TV07) effects on respiratory mortality in Bloemfontein at the 25th, 75th, and 99th percentiles. Points indicate relative risks (RR), vertical lines are 95% confidence intervals, and the dashed line represents no effect (RR = 1). Analysis varied spline degrees of freedom (3–6) and lag durations (14,21,28 days).


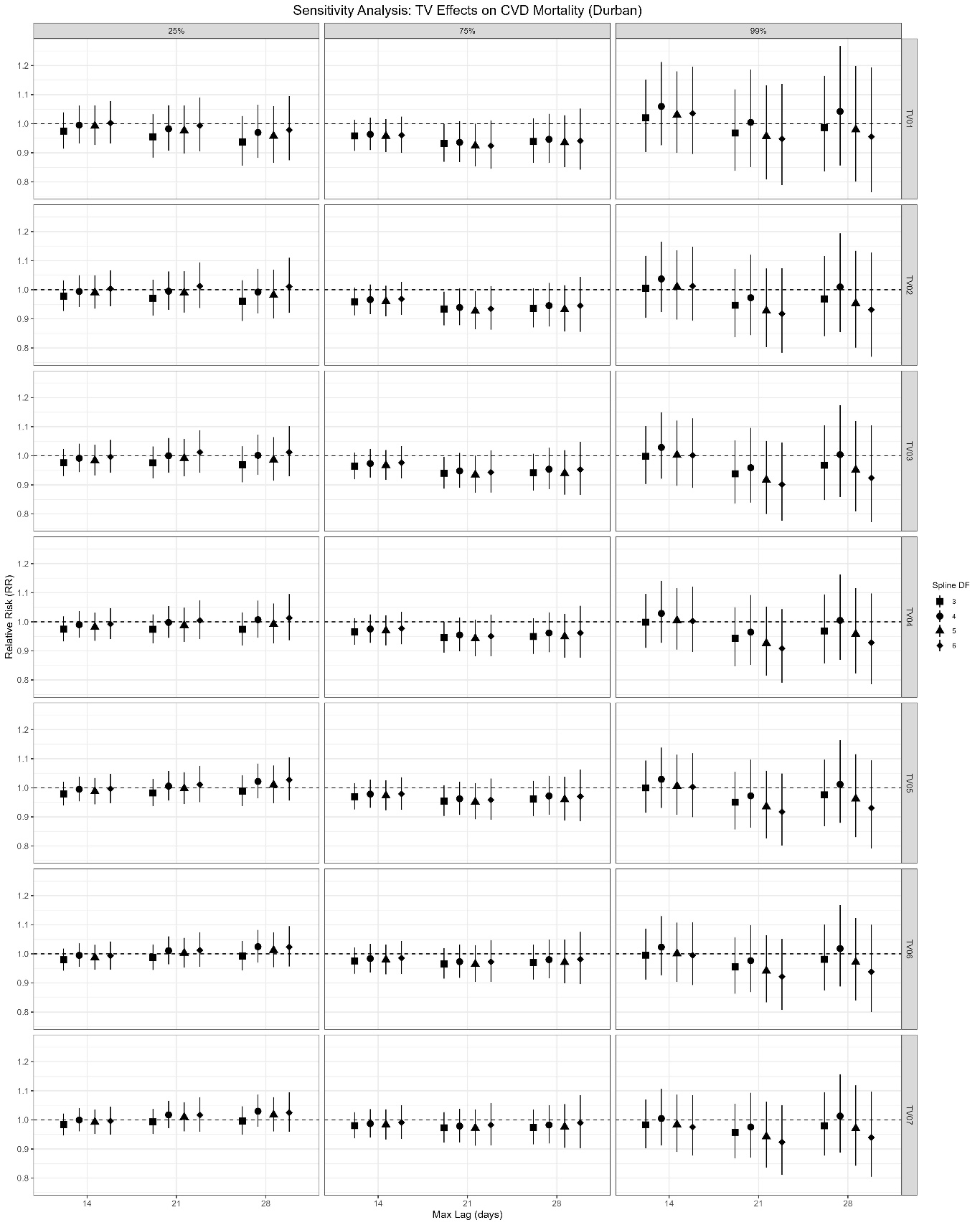


Figure A5. Sensitivity analysis of temperature variability (TV01–TV07) effects on cardiovascular mortality in Durban at the 25th, 75th, and 99th percentiles. Points indicate relative risks (RR), vertical lines are 95% confidence intervals, and the dashed line represents no effect (RR = 1). Analysis varied spline degrees of freedom (3–6) and lag durations (14,21,28 days).


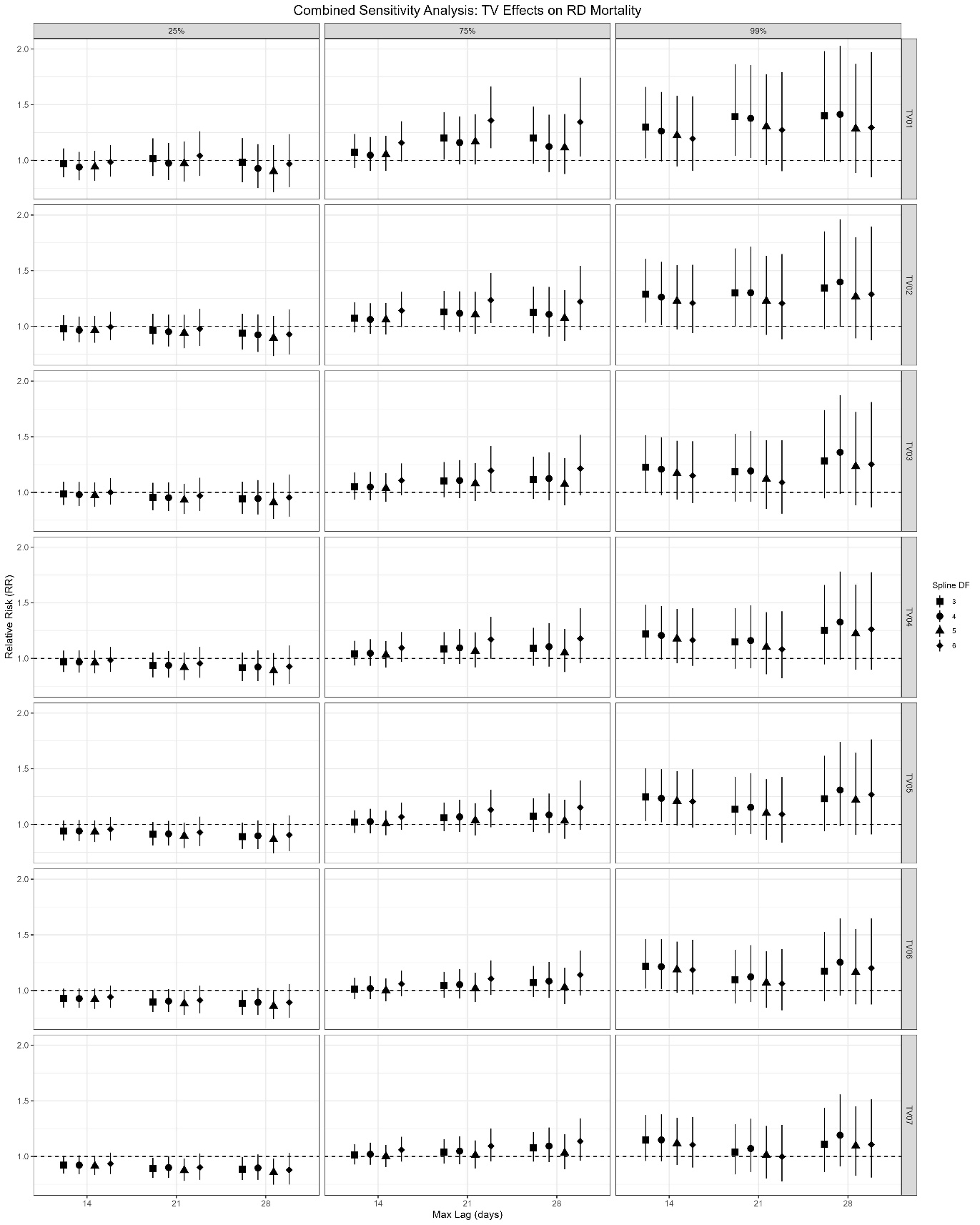


Figure A6. Sensitivity analysis of temperature variability (TV01–TV07) effects on respiratory mortality in Durban at the 25th, 75th, and 99th percentiles. Points indicate relative risks (RR), vertical lines are 95% confidence intervals, and the dashed line represents no effect (RR = 1). Analysis varied spline degrees of freedom (3–6) and lag durations (14,21,28 days).


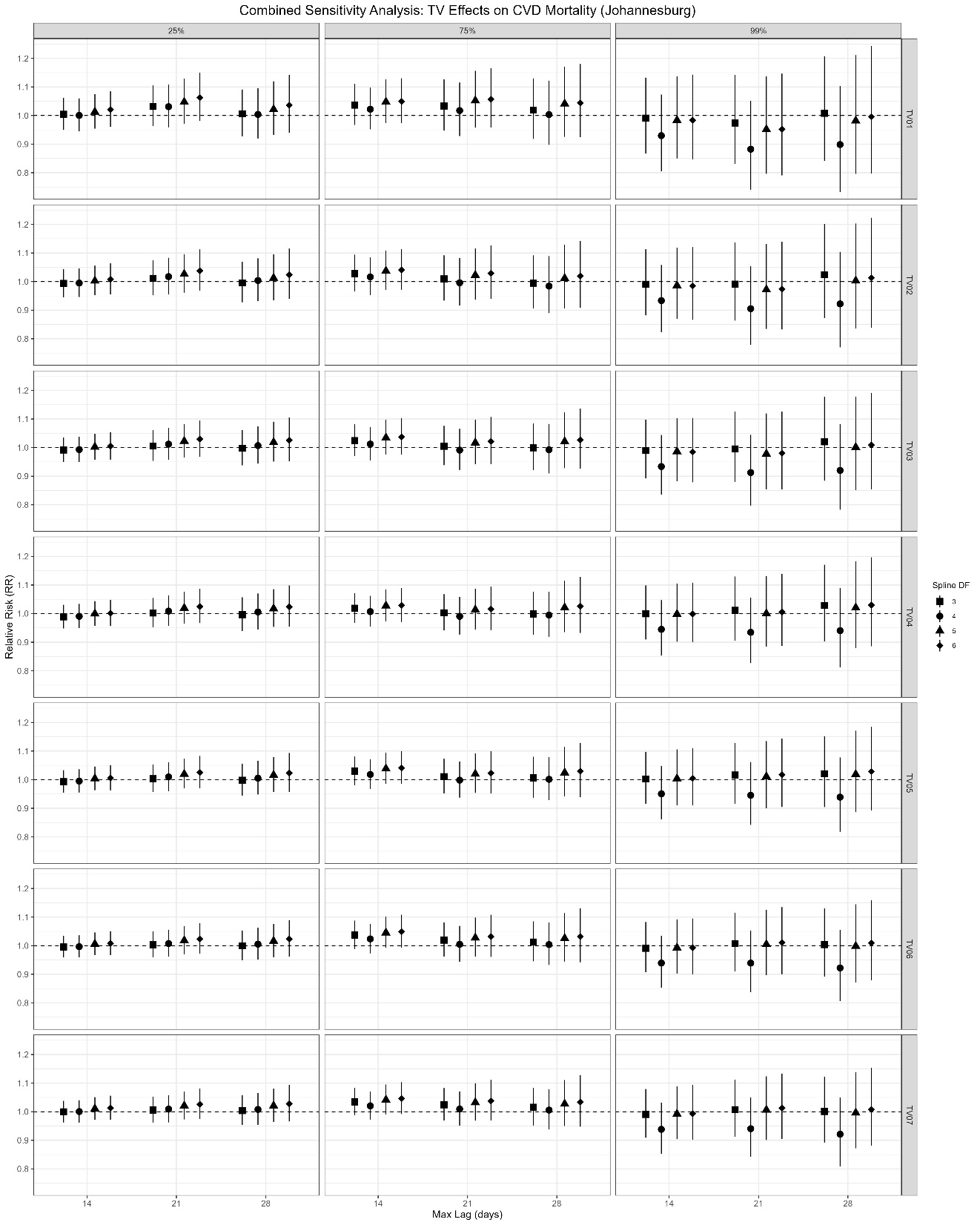


Figure A7. Sensitivity analysis of temperature variability (TV01–TV07) effects on cardiovascular mortality in Johannesburg at the 25th, 75th, and 99th percentiles. Points indicate relative risks (RR), vertical lines are 95% confidence intervals, and the dashed line represents no effect (RR = 1). Analysis varied spline degrees of freedom (3–6) and lag durations (14,21,28 days).


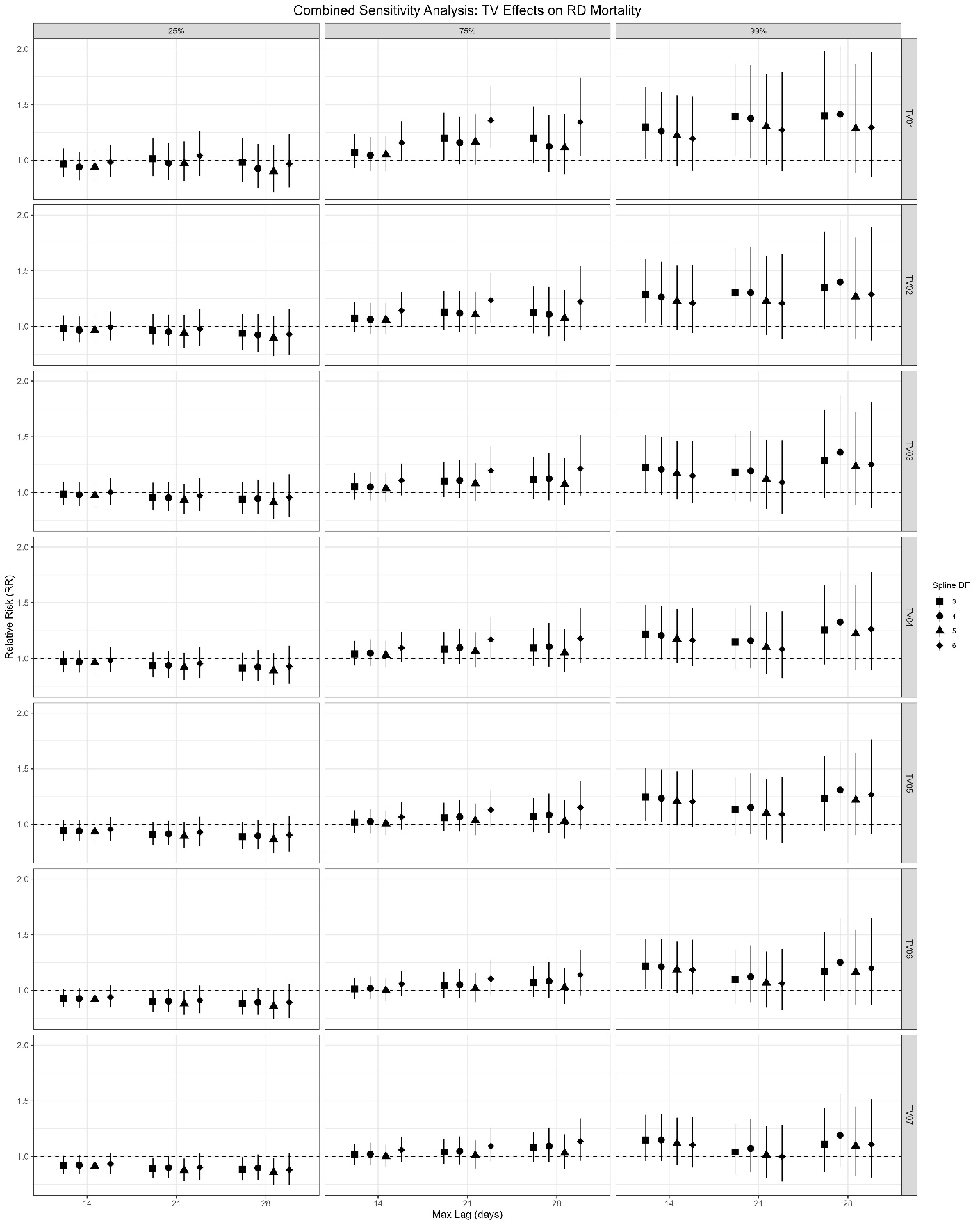


Figure A8. Sensitivity analysis of temperature variability (TV01–TV07) effects on respiratory mortality in Johannesburg at the 25th, 75th, and 99th percentiles. Points indicate relative risks (RR), vertical lines are 95% confidence intervals, and the dashed line represents no effect (RR = 1). Analysis varied spline degrees of freedom (3–6) and lag durations (14,21,28 days).


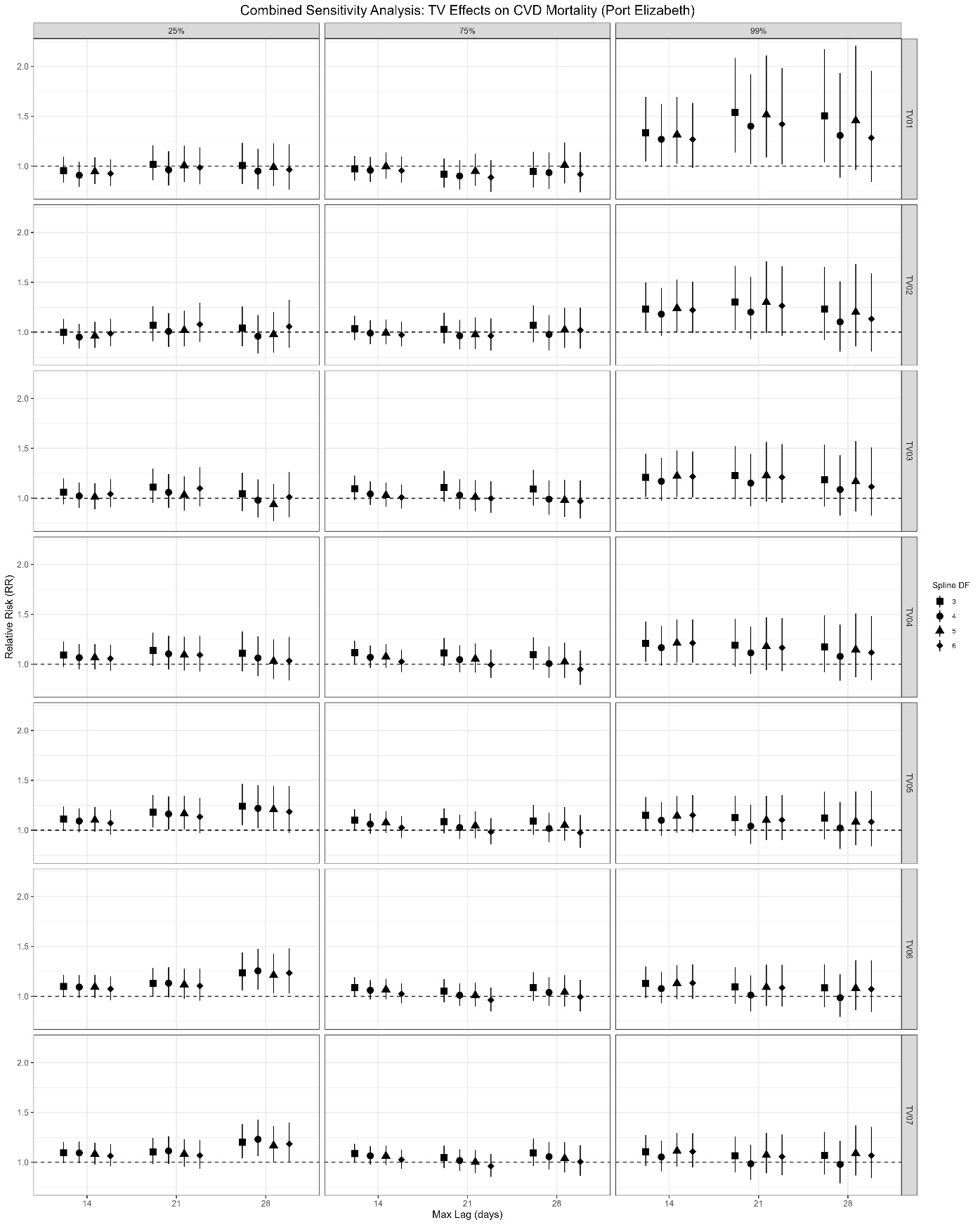


Figure A9. Sensitivity analysis of temperature variability (TV01–TV07) effects on cardiovascular mortality in Port Elizabeth at the 25th, 75th, and 99th percentiles. Points indicate relative risks (RR), vertical lines are 95% confidence intervals, and the dashed line represents no effect (RR = 1). Analysis varied spline degrees of freedom (3–6) and lag durations (14,21,28 days).


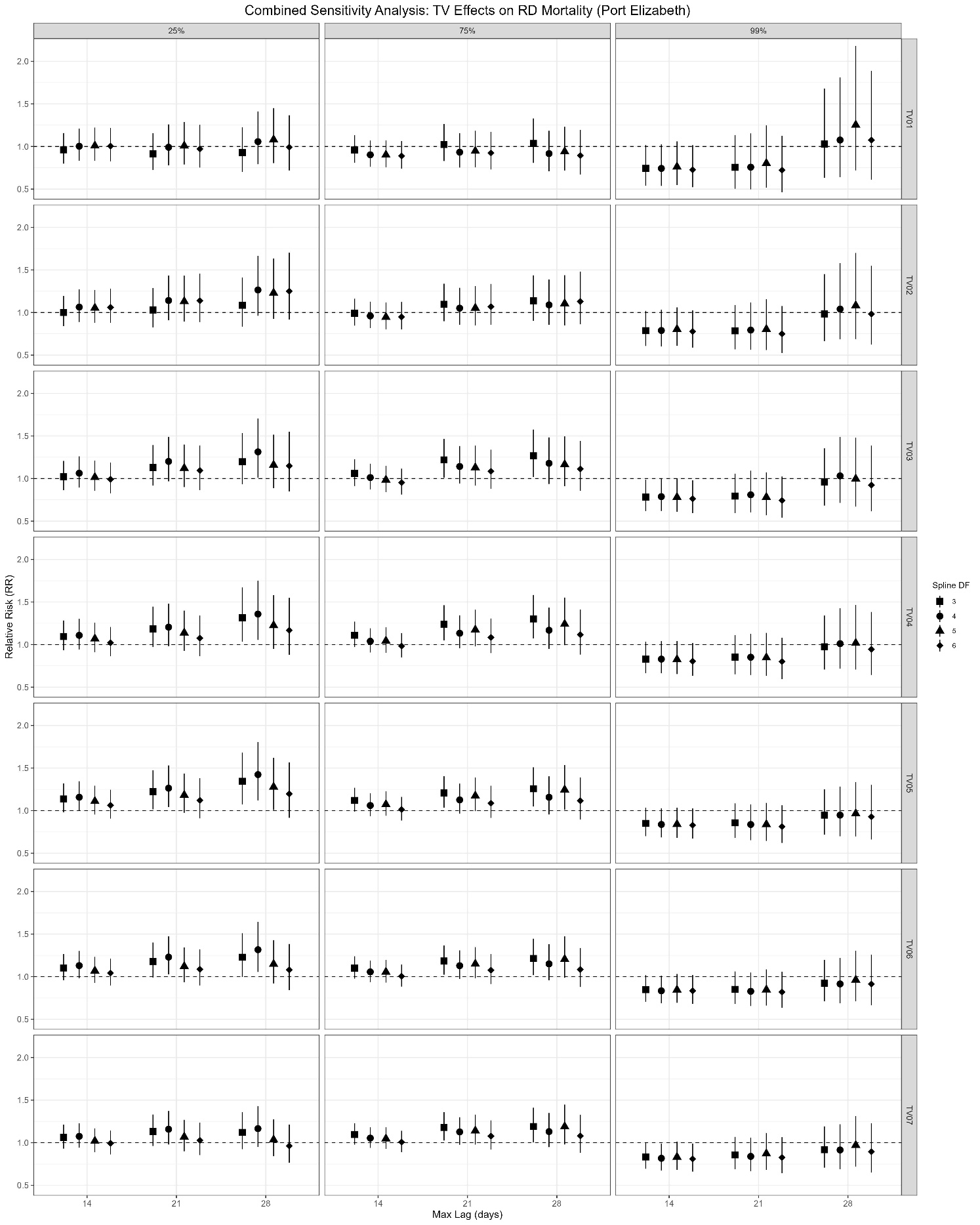


Figure A10. Sensitivity analysis of temperature variability (TV01–TV07) effects on respiratory mortality in Port Elizabeth at the 25th, 75th, and 99th percentiles. Points indicate relative risks (RR), vertical lines are 95% confidence intervals, and the dashed line represents no effect (RR = 1). Analysis varied spline degrees of freedom (3–6) and lag durations (14,21,28 days).
